# Grandmother's pregnancy complications and autism spectrum disorders in grandchildren, a California multigenerational cohort study

**DOI:** 10.1002/jcv2.70047

**Published:** 2025-09-05

**Authors:** Ting Chow, Qi Meng, Jingyuan Xiao, Karl O’Sharkey, Zeyan Liew, Beate Ritz

**Affiliations:** ^1^ Department of Epidemiology Fielding School of Public Health University of California, Los Angeles Los Angeles California USA; ^2^ Department of Environmental Health Sciences Yale School of Public Health New Haven Connecticut USA; ^3^ Yale Center for Perinatal, Pediatric, and Environmental Epidemiology Yale School of Public Health New Haven Connecticut USA; ^4^ Department of Environmental Health Sciences Fielding School of Public Health University of California, Los Angeles Los Angeles California USA; ^5^ Department of Neurology Geffen School of Medicine University of California, Los Angeles Los Angeles California USA

**Keywords:** autism spectrum disorders, grandparents, mothers

## Abstract

**Background:**

Pregnancy complications have been associated with offspring autism spectrum disorders (ASD). There has also been increasing evidence for multigenerational risk factors of ASD.

**Methods:**

In a multigenerational California birth cohort of 1,740,379 mother–child pairs, we investigated pregnancy complications when the grandmother was pregnant with the mother's generation and their associations with ASD in grandchildren. We estimated odds ratios (ORs) and 95% CIs using logistic regression.

**Results:**

The odds of ASD in grandchildren were higher among grandmothers who experienced pregnancy hypertensive disorders (OR, 1.23; 95% CI, 1.12–1.36) or infections (OR, 1.14; 95% CI, 1.01–1.28) compared to grandmothers who did not have the respective condition in pregnancy. The odds of ASD in grandchildren were also higher among mothers born prematurely (OR, 1.06; 95% CI, 1.02–1.11) compared to mothers born at term and elevated among mothers born with low birth weight (OR, 1.05; 95% CI, 1.00–1.11) compared to mothers with normal birth weight.

**Conclusions:**

ASD risk in grandchildren is higher among grandmothers who experienced hypertensive disorders or infections when pregnant with their daughters. Future studies are needed to explore the biologic underpinnings of possible multigenerational mechanisms.

## Key Points

**What's known?**

- Pregnancy complications such as hypertensive disorders and infections have been associated with higher ASD risk in offspring.

**What's New?**

- Hypertensive disorders and infections when the grandmother is pregnant with her daughter may be associated with higher ASD risk in grandchildren.

**What's relevant?**

- With the prevalence of hypertensive disorders and infections rising, it is important to understand potential multi‐generational health impacts.
- Future studies with longer follow‐up times, more detailed pregnancy complication information, and/or epigenetic measures are needed to replicate and expand on these findings.

## INTRODUCTION

Autism spectrum disorders (ASD) is a neurodevelopmental disorder mainly defined by difficulties in social communication, repetitive behaviors, and restricted interests manifesting in early childhood (American Psychiatric Association, 2013; Centers for Disease Control and Prevention, 2020). The estimated prevalence of ASD in 2020 was 27.6 per 1000 children in the United States and 44.9 per 1000 children in California (among children born in 2012) according to the Centers of Disease Control and Prevention's (CDC) Autism and Developmental Disabilities Monitoring network (Maenner et al., 2023). While ASD is multifactorial, apart from genetics and older age at pregnancy, maternal risk factors for ASD include the mother's pregnancy complications including hypertensive disorders (Dachew et al., 2018; Maher et al., 2018), infections (Jiang et al., 2016), and diabetes (Xu et al., 2014). In the United States, the rates of hypertensive disorders and diabetes in pregnancy have been increasing in the past decades (Bornstein et al., 2020). In addition, according to the CDC, the rates of infections such as chlamydia, gonorrhea, and syphilis in pregnancy have also increased by 2%, 16%, and 34% respectively from 2016 to 2018 (Gregory & Ely, 2020).

Pregnancy complications may lead to suboptimal in utero environments that may impair fetal neurodevelopment. Maternal hypertensive disorders and diabetes are thought to lead to placental dysfunction, fetal hypoxia, and/or increased oxidative stress (Maher et al., 2018; Xu et al., 2014). Infections in pregnancy may affect offspring neurodevelopment through actions of the pathogen themselves or triggering a harmful immune response of the mother to the pathogen (Jiang et al., 2016). Maternal pregnancy complications may also lead to adverse birth outcomes (Bromfield et al., 2023; Cram et al., 2002; Farrar et al., 2016), such as preterm birth (PTB) and/or low birth weight (LBW), which in turn are risk factors for ASD (Guo et al., 2022).

There is increasing evidence for multigenerational risk factors of ASD. Recent studies have reported elevated risks of ASD among children whose grandparents were of younger or older age (Frans et al., 2013; Gao et al., 2020; Golding et al., 2010) and whose grandmothers smoked while pregnant with the mothers (Golding et al., 2017). In addition, one study reported higher ASD risk among children whose mothers or fathers were born prematurely or were LBW (Xiao et al., 2021). Germ cell reprogramming is one proposed mechanism for multigenerational transmission of disease where epigenetic markers typically removed and reestablished in early fetal development are impaired or altered (Bohacek & Mansuy, 2015; Mouat & LaSalle, 2022). It has been hypothesized that maternal immune activation (MIA) may have multigenerational impacts through epigenetic modifications and germ cell‐dependent inheritance (Pollak & Weber‐Stadlbauer, 2020).

A study conducted in a population of predominately European ancestry (Xiao et al., 2021) reported an increased risk of ASD in grandchildren whose mothers or fathers were born prematurely or were LBW. However, less is known about multigenerational associations of pregnancy complications in the grandmother and ASD in grandchildren. Here, we investigate the associations between pregnancy complications (hypertensive disorders, infection, and diabetes) experienced by the grandmother and ASD in her grandchildren and assess whether these associations are mediated by grandmothers giving birth to their daughter (the mother's generation) prematurely. We hypothesize that among grandmothers who experienced hypertensive disorders, infections, or diabetes in pregnancy, grandchildren are at higher odds of ASD compared to grandmothers without these respective conditions. Additionally, we hypothesize that these associations may be partially mediated by the mother's premature birth which may have disrupted her own development during the fetal growth period.

## METHODS

### Study population

This is a California population‐based multigenerational birth cohort study of children born between 2001 and 2019 to mothers who were born between 1983 and 2001. Briefly, ASD diagnostic records obtained from the California Department of Developmental Services (DDS) (Department of Developmental Services, 2023) and birth records obtained from the California Department of Public Health (California Department of Public Health, 2024) were probabilistically linked (using National Program of Cancer Registries Registry Plus Link Plus Software [U.S. Department of Health and Human Services & National Center for Chronic Disease Prevention and Health Promotion, 2023]) based on child and parental identifiers as previously described (Chow et al., 2024). The DDS Client Developmental Evaluation Report includes primary diagnosis of autistic disorder according to the Diagnostic and Statistical Manual of Mental Disorders, fourth edition, revised (DSM‐IV‐R) until 2014 or DSM‐V (Department of Developmental Services, 2015). California born children with a linked ASD diagnostic record from the DDS were considered children with ASD and those whose birth record did not link with an ASD diagnostic record were considered non‐cases.

Children and mothers were eligible for this study if they had been singleton births with plausible birth weight (500–6800 g) and gestational age (20–50 weeks), mother's and grandmother's ages were 18–55 years, and information on child's sex, grandmother's parity, race, ethnicity, and pregnancy complications were available. The final analytical cohort includes 1,740,379 mother–child pairs with complete information for analysis.

This study has been approved by the California State and UCLA Institutional Review Boards with waiver of informed consent since this is a data linkage only study.

### Statistical analysis

We first evaluated the associations between offspring ASD risk and grandmother's pregnancy complications that is, when pregnant with the mother. We performed logistic regression to obtain odds ratios (ORs) and 95% CIs, adjusting for child's birth year and sex (male, female), mother's birth year, grandmother's parity (1, 2, 3 or more births), grandmother's age (18–24, 25–29, 30–34, 35–55 years), and grandmother's race/ethnicity (Asian Pacific Islander, Black, Hispanic (any race), White). Covariates were included in the regression models as categorical (see above) or continuous (birth year). Grandmother's pregnancy complications (hypertensive disorders, infections, and diabetes in pregnancy) were recorded on maternal birth certificates as a string of 2‐digit pregnancy complication codes (Table S1). Grandmothers without the respective pregnancy complication were considered the reference group for each respective complication. All covariates and pregnancy complications were recorded on the mother or grandchild's birth certificates.

As sensitivity analyses, we additionally included grandmother's education (8th grade or less, 9th to 12th but no diploma, high school, some degree less than college, college or more) and mother's birth weight (<2500 g, ≥2500 but <4000 g, ≥4000 g) for the subset of mother–child pairs for whom the grandmother's educational level was available that is, starting with 1989 birth records. These additional covariates are used as proxies to adjust for some of the confounding by grandmother's smoking status, which has been reported to be associated with ASD (adjusted OR = 1.41) (Golding et al., 2017) but was not collected on the California birth records until more recent birth years. A recent study found that five variables recorded on the California birth records (child's birth year, child's birth weight, mother's race/ethnicity, parity, and education) were predictive of mother's smoking in pregnancy with an overall discrimination accuracy of 0.72 and an area under the receiver operating characteristic curve of 0.81 (He et al., 2024). We additionally report e‐values to quantify the minimum strength an uncontrolled confounder (such as grandmother's smoking) would need to exhibit with both grandmother's pregnancy complications and ASD risk in grandchildren to fully explain away the associations we observed (Mathur et al., 2018).

We then evaluated the associations between offspring ASD risk and the mother's adverse birth characteristics. The gestational week (according to the dates of last menses and of birth) when the mother was born was used to categorize PTB as being born <37 weeks gestation. We also investigated a subset of very PTB defined as being born <32 gestational weeks and used term birth (37+ weeks of gestation) as the reference group for all PTBs. Mother's birth weight is categorized as LBW (<2500 g), normal weight (≥2500 but <4000 g), and macrosomia (≥4000 g) and we will use normal birth weight as the reference group.

Finally, we additionally performed mediation analyses for mother's premature birth while estimating effects for each of the grandmother's pregnancy complications and ASD risk in grandchildren taking mother's PTB into account. Formally, casual mediation decomposition of the total effect (TE) requires identifiability (the stable unit treatment value assumption, consistency, conditional exchangeability, and positivity), and conditional exchangeability which requires (1) no uncontrolled confounding between the exposure and outcome, (2) no uncontrolled confounding between the mediator and outcome, (3) no uncontrolled confounding between the exposure and mediator, and (4) no exposure‐induced confounding of the mediator and outcome (Wang & Arah, 2015). To calculate the standard controlled direct effect (CDE), conditional exchangeability requirements (1) and (2) are sufficient.

Logistic regression was used to estimate the total effect between offspring ASD and each of the grandmother's pregnancy complications and to estimate the controlled direct effect had mothers not been preterm. The portion eliminated (PE) will equal the estimated total effect minus the estimated controlled direct effect. The PE divided by the estimated total effect will equal the proportion of the estimated effect of grandmother's pregnancy complication on ASD in grandchildren that is due to mediation by mother's having been preterm, the interaction between grandmother's pregnancy complication and mother's having been preterm, or both the mediation and the interaction. The corresponding bootstrap percentile 95% CIs were generated using 1000 bootstrap samples. To preserve statistical power, we categorized the child's (2001–2009, 2010–2013, 2014–2016, 2017–2019) and mother's (1983–1985, 1986–1988, 1989–1991, 1992–2001) birth years and adjusted for them using continuous variables. In a sensitivity analysis, we also instead relied on mothers very preterm status as the mediator since very premature birth is a more severe adverse birth outcome.

Data analysis was performed using SAS 9.4 (SAS Institute Inc.) and e‐values using the online calculator (Mathur et al., 2018).

## RESULTS

Our multigenerational study population included 1,740,379 mother–child pairs among whom 27,904 (1.6%) children were diagnosed with ASD (Table 1). While 51.1% of this birth cohort consisted of male children, 78.5% of the ASD cases were male children. About one third (31.8%) of the mothers were the firstborn daughters of the grandmothers and most grandmothers had given birth to their daughter by the age of 30 years. A majority of grandmothers were of Hispanic ethnicity of any race (50.9%) followed by grandmothers of White race (35.1%).

**TABLE 1:** Study population characteristics by autism spectrum disorders.

Study population characteristics	Case (*N* = 27,904)	Total (*N* = 1,740,379)
Child's sex, *n* (%)		
Female	6004 (21.5%)	851,199 (48.9%)
Male	21,900 (78.5%)	889,180 (51.1%)
Mother's birth year, mean (SD)	1988.6 (4.00)	1988.3 (3.94)
Grandmother's parity, *n* (%)		
1	9067 (32.5%)	553,109 (31.8%)
2	8407 (30.1%)	538,310 (30.9%)
3 or more	10,430 (37.4%)	648,960 (37.3%)
Grandmother's race and ethnicity, *n* (%)		
Asian Pacific Islander	1297 (4.6%)	92,390 (5.3%)
Black	2611 (9.4%)	151,513 (8.7%)
Hispanic	15,203 (54.5%)	886,413 (50.9%)
White	8793 (31.5%)	610,063 (35.1%)
Grandmother's age categories, *n* (%)		
18–24 years	12,627 (45.3%)	781,853 (44.9%)
25–29 years	8354 (29.9%)	534,252 (30.7%)
30–34 years	4724 (16.9%)	297,673 (17.1%)
35–55 years	2199 (7.9%)	126,601 (7.3%)
Grandmother's hypertensive disorders in pregnancy, *n* (%)		
Yes	435 (1.6%)	21,953 (1.3%)
Grandmother's infections in pregnancy, *n* (%)		
Yes	293 (1.1%)	15,131 (0.9%)
Grandmother's diabetes in pregnancy, *n* (%)		
Yes	293 (1.1%)	16,480 (0.9%)
Mother's birthweight categories, *n* (%)		
Normal birthweight (≥2500 g but <4000 g)	24,035 (86.1%)	1,503,914 (86.4%)
Macrosomia (≥4000 g)	2491 (8.9%)	154,787 (8.9%)
Low birthweight (<2500 g)	1378 (4.9%)	81,678 (4.7%)
Mother's gestational age categories, *n* (%)		
Non‐preterm (≥37 weeks)	25,330 (90.8%)	1,588,935 (91.3%)
Preterm (<37 weeks)	2574 (9.2%)	151,444 (8.7%)

The odds of ASD differed by grandmother's pregnancy complications after adjusting for the child's birth year and sex, mother's birth year, grandmother's parity, age, and race/ethnicity (Table 2). The odds of ASD were higher among grandmothers who had experienced pregnancy hypertensive disorders (OR, 1.23; 95% CI, 1.12–1.36) or infections (OR, 1.14; 95% CI, 1.01–1.28) but not diabetes (OR, 1.06; 95% CI, 0.94–1.19) compared to grandmothers who did not suffer from the respective condition in pregnancy. Additional adjustment for grandmother's education and mother's birth weight did not change the results (Table S2). The e‐values to fully explain away the associations observed between grandmother's hypertensive disorders or infections in pregnancy with ASD in grandchildren are 1.76 and 1.54 respectively (Table S3).

**TABLE 2:** Grandmother's pregnancy complications and autism spectrum disorders in grandchildren.

	Children, *n*		Odds ratios (95% CI)	
	Cases	Total	Model 0	Model 1
Grandmother's hypertensive disorders in pregnancy				
No	27,469	1,718,426	Reference	Reference
Yes	435	21,953	1.24 (1.13–1.36)	1.23 (1.12–1.36)
Grandmother's infections in pregnancy				
No	27,611	1,725,248	Reference	Reference
Yes	293	15,131	1.15 (1.02–1.29)	1.14 (1.01–1.28)
Grandmother's diabetes in pregnancy				
No	27,611	1,723,899	Reference	Reference
Yes	293	16,480	1.07 (0.95–1.20)	1.06 (0.94–1.19)
Mothers born premature				
Non‐preterm (≥37 weeks)	25,330	1,588,935	Reference	Reference
Preterm (<37 weeks)	2574	151,444	1.08 (1.04–1.13)	1.06 (1.02–1.11)
Very preterm (<32 weeks)	354	18,459	1.22 (1.10–1.36)	1.19 (1.07–1.32)
Mothers birthweight				
Normal birthweight (≥2500 g but <4000 g)	24,035	1,503,914	Reference	Reference
Macrosomia (≥4000 g)	2491	154,787	1.01 (0.96–1.05)	1.02 (0.97–1.06)
Term macrosomia (≥4000 g and ≥37 weeks)	2397	149,018	1.00 (0.96–1.05)	1.02 (0.97–1.06)
Low birthweight (<2500 g)	1378	81,678	1.07 (1.01–1.13)	1.05 (1.00–1.11)
Term low birthweight (<2500 g and ≥37 weeks)	720	43,355	1.06 (0.98–1.14)	1.04 (0.97–1.12)

*Note*: Very preterm is a subset of preterm, term macrosomia is a subset of macrosomia, term low birth weight is a subset of low birth weight. Model 0 adjusts for offspring birth year (continuously scaled) and offspring sex (male, female participants). Model 1 adjusts for the variables in model 0 plus mother's birth year (continuously scaled), grandmother's parity (1, 2, 3, or more), grandmother's age (18–24, 25–29, 30–34, 35–55 years) and grandmother's race/ethnicity (Asian Pacific Islander, Black, Hispanic, White).

The odds of ASD also differed by mother's adverse birth characteristics after adjusting for the child's birth year and sex, mother's birth year, grandmother's parity, age, and race/ethnicity (Table 2). The odds of ASD were higher among mothers born prematurely (OR, 1.06; 95% CI, 1.02–1.11) and very prematurely (OR, 1.19; 95% CI, 1.07–1.32) compared to mothers being born at term, respectively. The odds of ASD were similar among mothers born with a macrosomic birth weight (OR, 1.02; 95% CI, 0.97–1.06) and slightly elevated among mothers born with LBW (OR, 1.05; 95% CI, 1.00–1.11) compared to mothers with normal birth weight, respectively.

We did not observe mediating effects by mothers born prematurely on the associations between grandmother's hypertensive disorders or infection and ASD in grandchildren (Table S4).

## DISCUSSION

In this large and diverse California birth cohort covering almost 40 years, we observed that the risk of ASD in grandchildren is elevated among grandmothers with hypertensive disorders or infections in pregnancy compared to grandmothers who did not suffer from these respective pregnancy complications. With the rising prevalence of hypertensive disorders and infection in pregnancy (Bornstein et al., 2020; Gregory & Ely, 2020), these results are of public health importance as they suggest these pregnancy complications may affect neurodevelopmental outcomes two generations later.

Results remained essentially the same even after adjusting for grandmother's education and mother's birth weight or preterm status. However, grandmother's smoking is a potential confounder that we could not directly adjust. A study from the United Kingdom reported an association between grandmother's smoking in pregnancy and ASD in grandchildren (adjusted OR = 1.41) (Golding et al., 2017). Yet, the calculated e‐values compared with the estimate of grandmother's smoking and ASD risk in grandchildren previously reported indicate that confounding by grandmother's smoking is unlikely to fully explain away the associations of grandmother's hypertensive disorders or of infections with ASD occurrence in the grandchildren. While there is some evidence that maternal smoking may be related to maternal hypertensive disorders in pregnancy (Wang et al., 2022), it is less likely that maternal smoking would be related to maternal infections. Therefore, grandmother's smoking might not be a confounder between the association of grandmother's infection in pregnancy and ASD risk in grandchildren. In addition, the majority of grandmothers in our California multigenerational cohort are Hispanics of any race. A study of children born in Los Angeles County found that Latina mothers reported very low smoking rates—lower than White mothers (Hoggatt et al., 2012).

However, there may still be residual confounding from other unmeasured potential confounders such as grandmother's behaviors such as drinking or smoking or her mental health. Measurement error of covariates may also result in residual confounding as the confounders we adjusted for were all recorded on the California birth certificates and are self‐reported. However, as these variables are collected on the mother's birth certificate well before ASD diagnosis of grandchildren, the measurement error is expected to be non‐differential with respect to the outcome of interest. While non‐differential misclassification most likely causes effect attenuation, the full extent of mismeasured or misclassified confounders on effect estimation is complex and difficult to quantify (Ogburn & Vanderweele, 2013).

It is also possible that some pregnancy complications may be hereditary; that is, grandmother and mother may share underlying heritable risk factors that may also be potential transgenerational confounders or mediators. For instance, family history of hypertension is a risk factor for developing hypertension (Ahn & Gupta, 2017). In contrast, a grandmother's infection in pregnancy is unlikely to influence whether or not her daughter develops an infection in her pregnancy. Results did not change, however, when we adjusted for mother's pregnancy complications. Mediation analysis for mother's hypertensive disorders was not possible due to a lack of statistical power as only about 4% of mothers had hypertensive disorders in pregnancy. In addition, ASD is highly hereditary (Sandin et al., 2017; Wiśniowiecka‐Kowalnik & Nowakowska, 2019) and as much as there are shared genetic factors related to both pregnancy complications and ASD these may play a role in the associations we observed.

While the association between grandmother's diabetes in pregnancy and ASD in grandchildren was in a positive direction, it might not have reached statistical significance since there was both a small number of grandmothers with diabetes who also had a grandchild with ASD and the magnitude of the OR was weak. We therefore did not attempt mediation analyses for grandmother's diabetes in pregnancy. Future research with a larger number of grandmothers with diabetes, data to differentiate between different types of diabetes (e.g., gestational, type 1, or type 2), glucose control in pregnancy, and pre‐pregnancy body mass index are needed to better understand potential risk and mechanisms involved.

There is increasing evidence from animal models that MIA in pregnancy may have multigenerational effects (Pollak & Weber‐Stadlbauer, 2020). In murine models, exposure to polyinosinic:polycytidylic acid (poly(I:C)), an immunostimulant to mimic viral immune activation, in early pregnancy resulted in reduced sociability and increased fear‐related behaviors in the subsequent three generations (Weber‐Stadlbauer et al., 2017) and in mid‐pregnancy this exposure resulted in increased depressive‐like behaviors and some increased hippocampal miRNA expression in the offspring two generations later (Berger et al., 2018).

Consistent with a Danish study, we found that ASD in grandchildren was increased among mothers born prematurely (or very prematurely) compared to mothers born at term and among mothers born LBW compared to mothers born with normal birth weight (Xiao et al., 2021). Proposed mechanisms for multigenerational transmission of disease include fetal germ cell reprogramming, where disturbances disrupt the establishment of epigenetic marks in the developing fetal germline (Bohacek & Mansuy, 2015; Mouat & LaSalle, 2022). Therefore, we investigated whether grandmother's pregnancy complications influenced grandchildren's ASD risk and whether this risk was mediated by the mothers having been preterm infants (as an indicator of disrupted fetal development).

We did not detect any evidence for mediation of grandchildren's ASD risk according to grandmother's hypertensive disorders or infection by mothers' preterm status. The CDEs also appeared elevated compared to the TEs, indicating that the associations between grandmother's hypertensive disorders or infections with ASD in grandchildren are stronger among mothers born term. A possible explanation for these results include collider stratification bias since the proportions eliminated were in the inverse direction and generally null, which was contrary to expectations and reminiscent of the ‘birth weight paradox’ seen in the association between maternal smoking and lower infant mortality (VanderWeele, 2014). Using PTB as a mediator may have induced collider bias introduced through an unknown confounder of the association between maternal PTB and ASD in offspring. Specifically, if there are unmeasured common causes between mother's PTB and ASD in grandchildren, conditioning on mother's PTB may open a backdoor path between grandmother's pregnancy complications and ASD in grandchildren. When we instead used very PTB as the mediator (more severe and perhaps better indicator of fetal developmental disturbance), we found that the proportion eliminated was reduced to null, especially for grandmother's infection.

In addition, while it was more common that mothers were born prematurely than mothers having had LBW (Table 2), preterm status may not be a good or very specific indicator or proxy for maternal fetal developmental disturbances that affect epigenetic markers. Other possible explanations include a lack of statistical power due to the small number of grandmothers who had suffered from each of the pregnancy complications and had a grandchild with ASD as well as the relatively small number of mothers born preterm with a child with ASD. This is reflected in the wide 95% CIs for the proportions eliminated that for the most part crossed the null value of 0%. Like with any birth cohort, there may also be live birth bias (Liew et al., 2015) which may occur if the daughters of exposed grandmothers (with pregnancy complications) who would also have been at risk for being born preterm are more likely to not be born alive.

Strengths of this study include its California wide coverage and its ethnically diverse population, a relatively large sample size, and pregnancy complications having been recorded on the birth records eliminating recall bias since grandmother's information was recorded before the grandchild's ASD diagnosis. All children with or without ASD were also included in the analytical study population according to the same eligibility criteria. In addition, there is a high specificity (low false positive rate) of ASD since assessments are made by well‐trained multidisciplinary teams at the DDS (Department of Developmental Services, 2002). However, growing awareness of ASD may have influenced the sensitivity of ASD over the years, so we added birth year into the covariate adjustment.

However, despite our large sample size, we still may have lacked statistical power in the analysis of grandmother's diabetes and for detecting mediation. Alternatively, there may have been live birth bias both in the grandmother–mother and mother–child generation and/or collider bias introduced in the mediation analyses. In addition, we were unable to adjust for grandmother's smoking but performed additional sensitivity analyses and did not have sufficient sample size and/or detailed data to look at specific types of hypertensive disorders, infections, or diabetes. These pregnancy complications were also self‐reported and therefore may have been misclassified; however, this misclassification is unlikely to be related to ASD in grandchildren but may have resulted in an attenuation of our effect sizes. Mothers in our study population also had to be born in California, stay in California, and give birth in California. California birth certificate data became available electronically in 1983, therefore by default maternal age was younger. Future studies with longer follow‐up are needed to confirm these findings. Nationwide studies may also be needed to capture all mothers who left California except for those who emigrated from the United States. Selection bias induced by moving out of California may however be minimal if moving is not strongly related to pregnancy complications or ASD outcome. We also do not have epigenetic data from the mother of the ASD child to examine the hypothesis of epigenetic reprogramming.

## CONCLUSION

Our findings suggest that there may be multigenerational impacts of pregnancy complications in grandmothers that increase the risk of the grandchildren developing ASD. These findings are important since pregnancy complications such as hypertensive disorders are common in the obstetric population and the rates are rising. However, future studies to confirm these findings are needed.

## AUTHOR CONTRIBUTIONS

**Ting Chow**: Conceptualization; data curation; formal analysis; funding acquisition; investigation; methodology; project administration; resources; writing—original draft; writing—review and editing. **Qi Meng**: Data curation; project administration; resources; writing—review and editing. **Jingyuan Xiao**: Methodology; resources; writing—review and editing. **Karl O’Sharkey**: Project administration; resources; writing—review and editing. **Zeyan Liew**: Conceptualization; methodology; resources; writing—review and editing. **Beate Ritz**: Conceptualization; data curation; funding acquisition; investigation; methodology; project administration; resources; supervision; writing—original draft; writing—review and editing.

## CONFLICT OF INTEREST STATEMENT

Dr. Beate Ritz received funding for research grants unrelated to this manuscript from NIH, the California Air Resources Board, NASA, DOE, and the MJFox foundation and serves as an expert witness for plaintiffs in a law suit related to contaminated baby food and ASD. Dr. Zeyan Liew received funding for research grants unrelated to this manuscript from the NIH. All other authors declared no biomedical financial interests or potential conflict of interests.

## ETHICAL CONSIDERATIONS

This study has been approved by the California State and UCLA Institutional Review Boards with waiver of informed consent since this is a data linkage only study. IRB: CPHS IRB. Study Name: Evaluating the Effects of Exposure to Air Pollution on Adverse Birth Outcomes in Southern California. Number: 12‐10‐0861. Initial Approval: 12/7/2012. Most recent continuing review approval: 10/3/2025 (Expiration 10/2/2026). Original amendment for the multigenerational component: Approved on 10/18/2021. Additional amendment for more DDS data: Approved on 1/11/2023. Note: This manuscript is covered under the amendments approved on 10/18/2021 and 1/11/2023. IRB: UCLA IRB. Study Name: EPOS (Environment Pregnancy and Outcomes Study). Number: IRB#10‐000617. Initial Approval: 9/9/2010. Most recent continuing review approval: 1/30/2025 (Expiration 1/29/2026)—CR number IRB‐10‐0617‐CR‐017. Original amendment for the multigenerational component: Approved 6/6/2022—Amendment number 10‐000617‐AM‐00018. Additional amendment for more DDS data: Approved 2/2/2023—Amendment number 10‐000617‐AM‐00019. Note: This manuscript is covered under the amendment numbers 10‐000617‐AM‐00018 (approved 6/6/2022) and 10‐000617‐AM‐00019 (approved 2/2/2023).

## Supporting information

Supplementary Material

## Data Availability

We are not permitted to share California birth data, but California birth certificates may be requested from the California Department of Public Health and diagnostic records from the California Department of Developmental Services.
