# Supplementary material for: Grandmother's pregnancy complications and autism spectrum disorders in grandchildren, a California multigenerational cohort study
Source: JCPP Adv. 2025 Sep 5;6(2):e70047. doi: 10.1002/jcv2.70047 (PMC13260705; doi:10.1002/jcv2.70047)
Supplement: Supplementary file 1 — Supplementary Material [file JCV2-6-e70047-s001.docx]

**Supplemental Tables**

Table S1. Hypertensive Disorders, Infections, and Diabetes in Pregnancy Codes from 1983-2001 Birth Records

|  | Mother's Birth Year | | |  |  |  |  |  |  |  |  |  |  |  |  |  |  |  |  |
| --- | --- | --- | --- | --- | --- | --- | --- | --- | --- | --- | --- | --- | --- | --- | --- | --- | --- | --- | --- |
|  | 1983 | 1984 | 1985 | 1986 | 1987 | 1988 | 1989 | 1990 | 1991 | 1992 | 1993 | 1994 | 1995 | 1996 | 1997 | 1998 | 1999 | 2000 | 2001 |
| Hypertensive Disorders |  |  |  |  |  |  |  |  |  |  |  |  |  |  |  |  |  |  |  |
| Pre-eclampsia/Eclampsia/Toxemia | x | x | x | x | x | x |  |  |  |  |  |  |  |  |  |  |  |  |  |
| Pre-eclampsia/Pregnancy-induced hypertension |  |  |  |  |  |  | x | x | x | x | x | x | x | x | x | x | x | x | x |
| Eclampsia |  |  |  |  |  |  | x | x | x | x | x | x | x | x | x | x | x | x | x |
| Chronic hypertension |  |  |  |  |  |  | x | x | x | x | x | x | x | x | x | x | x | x | x |
| Infections |  |  |  |  |  |  |  |  |  |  |  |  |  |  |  |  |  |  |  |
| Urinary Tract/Kidney Infection | x | x | x | x | x | x |  |  |  |  |  |  |  |  |  |  |  |  |  |
| Syphilis | x | x | x | x | x | x |  |  |  |  |  |  |  |  |  |  |  |  |  |
| Rubella | x | x | x | x | x | x | x | x | x | x | x | x | x | x | x | x | x | x | x |
| Hepatitis B |  |  |  |  |  |  | x | x | x | x | x | x | x | x | x | x | x | x | x |
| Kidney infection/Pyelonephritis |  |  |  |  |  |  | x | x |  |  |  |  |  |  |  |  |  |  |  |
| Pyelonephritis |  |  |  |  |  |  |  |  | x | x | x | x | x | x | x | x | x | x | x |
| Genital herpes |  |  |  |  |  |  |  |  | x | x | x | x | x | x | x | x | x | x | x |
| Other sexually transmitted disease |  |  |  |  |  |  |  |  | x | x | x | x | x | x | x | x | x | x | x |
| Diabetes | x | x | x | x | x | x | x | x | x | x | x | x | x | x | x | x | x | x | x |

Table S2. Grandmother's Pregnancy Complications and Autism Spectrum Disorders in Grandchildren, among mothers born 1989-2001 and non-missing Grandmother's Education

|  | **Children, n** | | **Odds Ratios (95% CI)** | |  |  |
| --- | --- | --- | --- | --- | --- | --- |
| **Grandmother's Hypertensive Disorders in Pregnancy** | **Cases** | **Total** | **Model 0** | **Model 1** | **Model 2** | **Model 3** |
| No | 12983 | 737098 | Reference | Reference | Reference | Reference |
| Yes | 226 | 10525 | 1.22 (1.07-1.40) | 1.22 (1.07-1.40) | 1.22 (1.07-1.40) | 1.21 (1.06-1.39) |
| **Grandmother's Infections in Pregnancy** | **Cases** | **Total** | **Model 0** | **Model 1** | **Model 2** | **Model 3** |
| No | 12973 | 736075 | Reference | Reference | Reference | Reference |
| Yes | 236 | 11548 | 1.14 (1.00-1.30) | 1.16 (1.01-1.32) | 1.16 (1.01-1.32) | 1.16 (1.01-1.32) |
| **Grandmother's Diabetes in Pregnancy** | **Cases** | **Total** | **Model 0** | **Model 1** | **Model 2** | **Model 3** |
| No | 13016 | 737617 | Reference | Reference | Reference | Reference |
| Yes | 193 | 10006 | 1.07 (0.93-1.24) | 1.07 (0.92-1.23) | 1.07 (0.92-1.23) | 1.06 (0.92-1.23) |

Model 0 adjusts for offspring birth year (continuously scaled) and offspring sex (male, female participants).

Model 1 adjusts for the variables in model 0 plus mother's birth year (continuously scaled), grandmother's parity (1, 2, 3 or more), grandmother's age (18-24, 25-29, 30-34, 35-55 years) and grandmother's race/ethnicity (Asian Pacific Islander, Black, Hispanic, White).

Model 2 adjusts for the variables in model 1 plus grandmother's education (8th grade or less, 9th to 12th but no diploma, high school, some degree less than college, college or more) and mother's birth weight (< 2500 grams, ≥ 2500 but < 4000 grams, ≥ 4000 grams).

Model 3 adjusts for the variables in model 1 plus grandmother's education (8th grade or less, 9th to 12th but no diploma, high school, some degree less than college, college or more) and mother's premature birth (<37 weeks, >=37 weeks).

Table S3. E-values to Explain away Grandmother's Pregnancy Complications and Autism Spectrum Disorders in Grandchildren

| **Pregnancy Complication** | **Odds Ratios (95% CI)** | **E-value** |
| --- | --- | --- |
| Grandmother's Hypertensive Disorders in Pregnancy | 1.23 (1.12-1.36) | 1.76 |
| Grandmother's Infections in Pregnancy | 1.14 (1.01-1.28) | 1.54 |

Note: https://www.evalue-calculator.com/evalue/

Table S4. Grandmother's Pregnancy Complications and Autism Spectrum Disorders in Grandchildren, by Mother's Premature Birth

|  |  | **Odds Ratios (Bootstrap Percentile 95% CI)** | |  |
| --- | --- | --- | --- | --- |
| **Grandmother's Pregnancy Complication** | **Mother's Premature Birth** | **Total Estimated Effect** | **Estimated Controlled Direct Effect** | **Proportion Eliminated, % (95% CI)** |
| Grandmother's Hypertensive Disorders | Mothers Born Preterm (< 37 weeks) | 1.23 (1.11-1.35) | 1.24 (1.11-1.37) | -4.8 (-32.6,22.9) |
| Grandmother's Hypertensive Disorders | Mothers Born Very Preterm (< 32 weeks) | 1.24 (1.10-1.37) | 1.24 (1.11-1.38) | -2.2 (-13.1,10.0) |
| Grandmother's Infections | Mothers Born Preterm (< 37 weeks) | 1.13 (1.01-1.27) | 1.18 (1.05-1.33) | -34.2 (-188.1,-0.2) |
| Grandmother's Infections | Mothers Born Very Preterm (< 32 weeks) | 1.18 (1.04-1.31) | 1.18 (1.04-1.32) | -3.1 (-18.2,7.6) |

Note: 95% CIs were obtained from 1000 bootstrap samples.

Associations are adjusted for offspring birth year (2001-2009, 2010-2013, 2014-2016, 2017-2019), offspring sex (male, female), mother's birth year (1983-1985, 1986-1988, 1989-1991, 1992-2001), grandmother's parity (1, 2, 3 or more), grandmother's age (18-24, 25-29, 30-34, 35-55 years) and grandmother's race/ethnicity (Asian Pacific Islander, Black, Hispanic, White).

The estimated controlled direct effect is the effect among mothers not born prematurely (>=37 weeks).
